# A Crosslinking Analysis of GAP-43 Interactions with Other Proteins in Differentiated N1E-115 Cells

**DOI:** 10.3390/ijms9091753

**Published:** 2008-09-16

**Authors:** Callise M. Ollom, John B. Denny

**Affiliations:** Department of Ophthalmology, University of Texas Health Science Center at San Antonio, San Antonio, Texas 78229, USA. E-Mail: cdenny@satx.rr.com (C. O.)

**Keywords:** Neuromodulin, cytoskeleton, filopodia, lipid rafts, palmitoylation, GAP-43, growth-associated protein of 43 kDa, DMSO, dimethyl sulfoxide, DSP, dithiobis (succinimidyl propionate), SDS, sodium dodecyl sulfate, CaM, calmodulin, DTT, dithiothreitol

## Abstract

It has been suggested that GAP-43 (growth-associated protein) binds to various proteins in growing neurons as part of its mechanism of action. To test this hypothesis *in vivo*, differentiated N1E-115 neuroblastoma cells were labeled with [^35^S]-amino acids and were treated with a cleavable crosslinking reagent. The cells were lysed in detergent and the lysates were centrifuged at 100,000 × g to isolate crosslinked complexes. Following cleavage of the crosslinks and analysis by two-dimensional gel electrophoresis, it was found that the crosslinker increased the level of various proteins, and particularly actin, in this pellet fraction. However, GAP-43 was not present, suggesting that GAP-43 was not extensively crosslinked to proteins of the cytoskeleton and membrane skeleton and did not sediment with them. GAP-43 also did not sediment with the membrane skeleton following nonionic detergent lysis. Calmodulin, but not actin or other proposed interaction partners, co-immunoprecipitated with GAP-43 from the 100,000 × g supernatant following crosslinker addition to cells or cell lysates. Faint spots at 34 kDa and 60 kDa were also present. Additional GAP-43 was recovered from GAP-43 immunoprecipitation supernatants with anti-calmodulin but not with anti-actin. The results suggest that GAP-43 is not present in complexes with actin or other membrane skeletal or cytoskeletal proteins in these cells, but it is nevertheless possible that a small fraction of the total GAP-43 may interact with other proteins.

## 1. Introduction

The growth-associated protein GAP-43 participates in neuronal development and is also present in certain regions of the normal adult brain, retina, and cornea [1–4]. During development and regeneration, axons must navigate correctly to their proper destination, and receptors on axonal growth cone filopodia bind various factors, including neurotrophins, netrins, semaphorins, ephrins, and slits, which results in the growth cone turning in the direction of the target [5]. GAP-43 is required in some way in this process, since retinal ganglion cell axons in mice that are deficient in this protein do not reach their target sites in the lateral geniculate nucleus [6].

Proteasome digestion studies suggested that GAP-43 is primarily unfolded at physiological ionic strength [7]. It is of interest how the protein might fold following its interaction with the membrane and with other proteins. GAP-43 becomes membrane-bound following its palmitoylation at Cys-3 and Cys-4 [8, 9] and this appears to be followed by the interaction of Arg-6, Arg-7, Lys-9, and Lys-13 with the lipid bilayer [10]. The protein remains membrane-bound if the palmitates are removed [10]. Protein palmitoylation is associated with neuronal growth [11] and a decrease in GAP-43 palmitoylation occurs as growth stops and synapses are formed [12]. NCAM (neural cell adhesion molecule)-mediated neurite outgrowth requires GAP-43 and causes the protein to be phosphorylated at Ser-41 by protein kinase C [13]. The increase in neuronal branching caused by the small GTPase Ral also results in the phosphorylation of GAP-43 by protein kinase C [14]. Ral is activated by laminin binding to integrin [14]. However, integrin-mediated outgrowth can also occur in the absence of GAP-43 [13].

It was suggested that GAP-43 might act by binding directly to F-actin, and *in vitro* studies demonstrated the binding of GAP-43 to existing F-actin [15] as well as binding when GAP-43 was present during actin polymerization [15–17]. In addition, *in vitro* experiments suggested that GAP-43 binds to spectrin [18]. GAP-43 has also been shown to enhance the binding of GTPγS to the α subunit of the heterotrimeric G protein Go [19]. GAP-43 has been proposed to bind directly to the synaptosomal associated protein SNAP-25 [20] and to participate in endocytosis via a direct interaction with the Rab5 effector protein rabaptin-5 [21]. In contrast, a yeast two-hybrid system showed that unphosphorylated GAP-43 interacted strongly only with calmodulin and a mutant which mimicked GAP-43 phosphorylated at Ser-41 did not interact with actin or other proteins [22]. GAP-43 binds to calmodulin *in vitro* [23], in synaptosomes [24], and *in vivo* [25]. This interaction is eliminated following the phosphorylation of GAP-43 by protein kinase C [23, 25]. The same basic region in GAP-43 (residues 39–56) that binds calmodulin has also been proposed to bind and sequester the acidic phospholipid phosphatidylinositol 4,5-bisphosphate (PI(4,5)P_2_) [26, 27].

The addition of a membrane permeable crosslinker to living, stimulated neurons is one approach to test for the direct interaction of GAP-43 with other proteins. In the present work crosslinking experiments, analyzed by immunoprecipitation and two-dimensional electrophoresis, were carried out on living, differentiated N1E-115 cells. It was found that GAP-43 did not sediment with crosslinked F-actin when crosslinker was added to these cells. It was also found that the crosslinker caused calmodulin, but not actin or other proposed interaction partners, to co-immunoprecipitate with GAP-43. Faint spots at 34 kDa and 60 kDa were also present in the immunoprecipitate when monoclonal anti-GAP-43 was used. The same results were obtained when cells were lysed in detergent prior to the addition of crosslinker, and GAP-43 did not sediment with the membrane skeleton when control or crosslinker-treated cells were lysed in nonionic detergent. The results suggest that the majority of GAP-43 in growing N1E-115 cells is not complexed to other proteins, but a small fraction of the total GAP-43 could nevertheless be involved in some protein-protein interactions.

## 2. Results and Discussion

### 2.1. Addition of DSP to living cells yields crosslinked complexes that will not enter a one-dimensional gel

The mouse neuroblastoma cell line N1E-115 was differentiated by the addition of 2% DMSO to the medium. After 4 days of this treatment, the cells displayed many neurites and growth cones. The membrane-permeable crosslinker dithiobis (succinimidyl propionate) (DSP), when added to these cultures, crosslinked actin and other proteins into large complexes with molecular weights too great for them to be resolved by SDS-PAGE.

**Figure 1.**
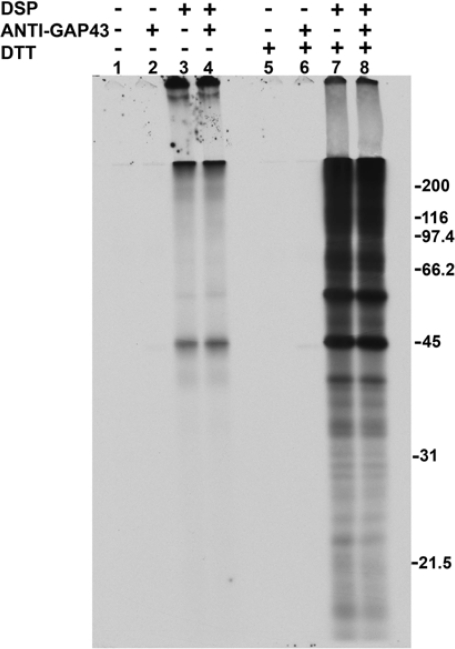
DSP treatment of N1E-115 cells yields crosslinked complexes that will not enter a one-dimensional gel. Molecular weight standards are shown at the right of each figure. Exposure time = 1 h.

**Figure 2.**
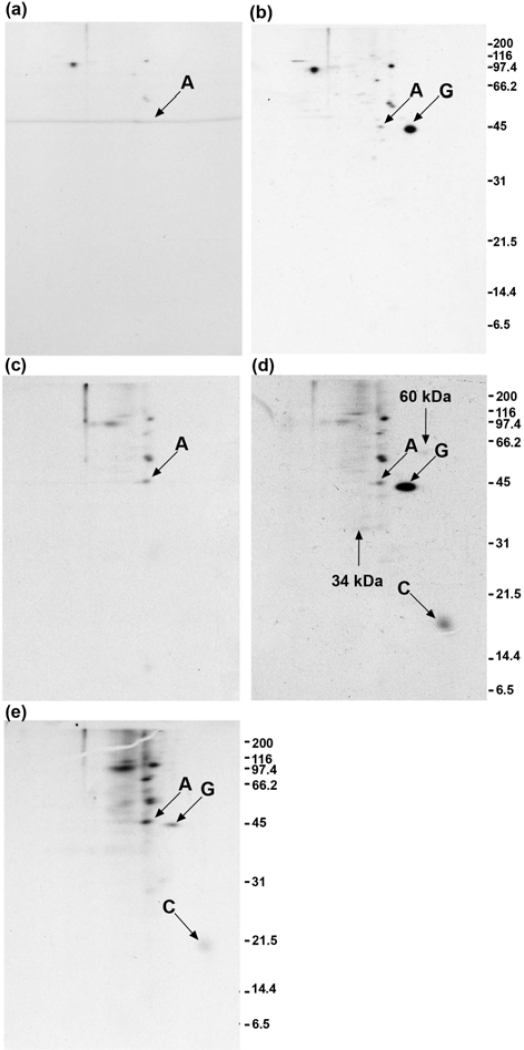
Calmodulin, but not actin, co-immunoprecipitates with GAP-43 following DSP treatment of cells. (a, b): DMSO. (c, d): DSP. Anti-β-galactosidase (a, c). Monoclonal anti-GAP-43 (b, d). (e) The immunoprecipitation supernatant from (d) was treated with monoclonal anti-calmodulin. Exposure time = 2 days (a–d) and 4 days (e).

In the experiment of Figure 1, differentiated N1E-115 cells were incubated with [^35^S]-labeled methionine and cysteine. The labeled cells received either DMSO alone as a control or DSP at a final concentration of 1 mM. This was followed by lysing the cells with SDS, addition of buffer containing NP-40 substitute, and shearing the lysates through a needle. The lysates were not centrifuged and received either monoclonal anti-GAP-43 or monoclonal anti β-galactosidase as a control, followed by incubation with protein G-agarose. The samples were divided and the material bound to the protein G-agarose was analyzed by SDS/PAGE either without DTT (lanes 1–4) or with DTT (lanes 5–8). The addition of DSP to cells caused the formation of large crosslinked complexes that associated with the agarose. Figure 1 shows that mainly actin, seen at 45 kDa, entered the gel in the absence of DTT (lanes 3 and 4) and that much more actin was present, as well as tubulin (seen at 55 kDa) if DTT were present (lanes 7 and 8). Immunoprecipitated GAP-43 migrated at 43 kDa and can be faintly detected, after only a 1 h exposure of the gel, both in the absence (lane 2) and presence (lane 6) of DTT. Longer exposure times under these immunoprecipitation conditions clearly showed the protein on this film as well as on the films of two-dimensional gels in Figure 2. The addition of anti-β-galactosidase yielded no band that migrated at 43 kDa in either the absence (lane 1) or presence (lane 5) of DTT. GAP-43 immunoprecipitation from DSP-treated cells could not be observed because of the high background seen in lanes 4 and 8.

### 2.2. Calmodulin, but not actin or other proposed interaction partners, co-immunoprecipitates with GAP-43 after addition of crosslinker to living cells

**Figure 3.**
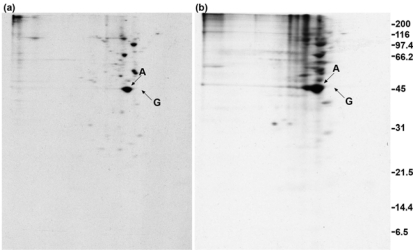
GAP-43 is not present in the 100,000 × g pellet fractions. (a) DMSO-treated cells. (b) DSP-treated cells. Exposure time = 4 h.

DMSO- and DSP-treated cells were lysed and sheared as in Figure 1, and the lysates were centrifuged at 100,000 × g. The supernatants were used for immunoprecipitation (Figure 2). Traces of actin and tubulin are present in the immunoprecipitates, which allowed these films to be precisely aligned with films of the pellet fractions seen in Figure 3. Control immunoprecipitates using anti-β-galactosidase were negative at the position of GAP-43, using cells that were either treated with DMSO (Figure 2a) or DSP (Figure 2c). GAP-43 (marked with a G) was immunoprecipitated by monoclonal anti-GAP-43 from DMSO-treated cells (Figure 2b) and from DSP-treated cells (Figure 2d). The acidic end of the gel is to the right for all two-dimensional gels. In Figure 2d, it is seen that DSP treatment of cells resulted in the co-immunoprecipitation of calmodulin (marked with a C) with GAP-43. In DMSO-treated cells there was no co-immunoprecipitation of calmodulin (Figure 2b), providing evidence that DSP caused the crosslinking of GAP-43 and calmodulin. By contrast, the level of actin (marked with an A) seen in Figure 2d resembles the level of background actin seen in Figures 2b and 2c. These results suggest that no GAP-43/actin crosslinked complexes were immunoprecipitated. Two faint spots at 34 kDa and 60 kDa are present in Figure 2d that are not present in Figures 2b or 2c, and they are each marked with an arrow. The supernatant obtained following GAP-43 immunoprecipitation in Figure 2d received anti-calmodulin, and the resulting immunoprecipitate is shown in Figure 2e. Additional GAP-43 was recovered when this was done. The levels of background actin and other background proteins are higher in Figure 2e than in Figure 2d because the film was exposed for a longer time.

**Figure 4.**
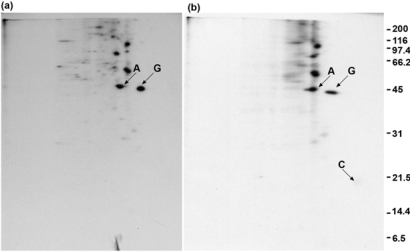
Polyclonal anti-GAP-43 yields co-immunoprecipitated calmodulin but not actin. (a) Cells treated with DMSO. (b) Cells treated with DSP. Exposure time = 6 days.

**Figure 5.**
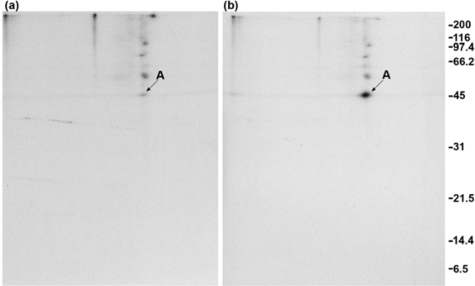
Anti-actin does not yield co-immunoprecipitated GAP-43 following DSP treatment of cells. (a) normal rabbit serum. (b) rabbit anti-actin. Exposure time = 2 days.

**Figure 6.**
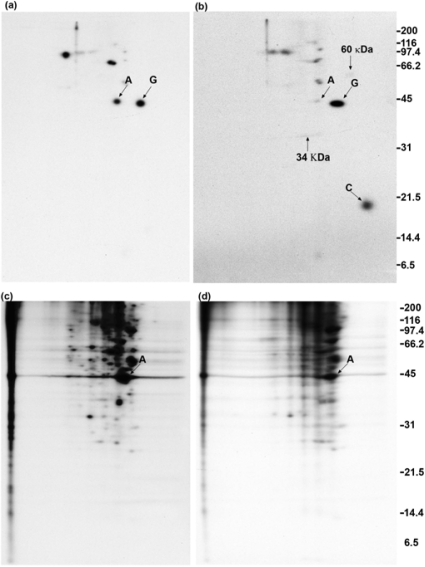
GAP-43 does not sediment with the membrane skeleton. Cells were treated with DMSO (a, c) or DSP (b, d). Anti-GAP-43 immunoprecipitates (a, b) and membrane skeleton pellets (c, d). Exposure times = 2 days (a, b) and 4 h (c, d).

**Figure 7.**
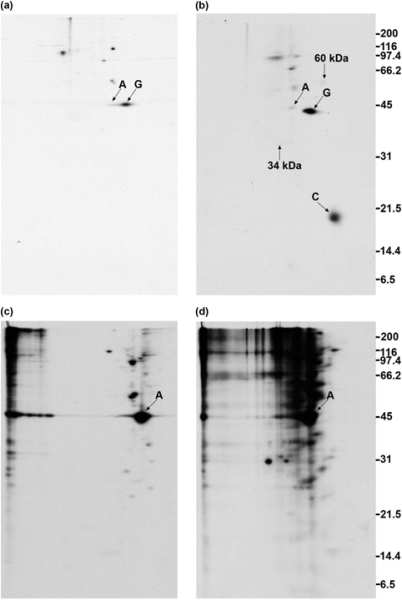
Addition of DSP to cell lysates. (a, c): DMSO. (b, d): DSP. Anti-GAP-43 immunoprecipitates (a, b) and pellet fractions (c, d). Exposure times = 2 days (a, b) and 4 h (c, d).

Immunoprecipitation of GAP-43 from membrane fractions of rat cerebellum growth cones yielded co-immunoprecipitated Goα, but only after GTPγS treatment of the membranes [28]. Two isoforms of Goα are expressed in DMSO-differentiated N1E-115 cells, and they each have a molecular weight of 39 kDa and isoelectric points of 5.8 and 5.55, respectively [29]. In Figure 2d, neither of these isoforms co-immunoprecipitated with GAP-43 following DSP treatment of cells. However, in the absence of crosslinker, a faint spot of about 40 kDa and with pI slightly more alkaline than β-actin (pI = 5.29) did appear in Figure 2b, and may represent co-immunoprecipitated Goα. This spot was absent from both the anti-β-galactosidase (Figure 2a) and normal rabbit serum (Figure 5a) controls. Proteins at the positions of the spectrin α-chain (285 kDa, pI = 5.21), spectrin β-chain (251 kDa, pI = 5.37), rabaptin-5 (99.5 kDa, pI = 4.97), and SNAP-25 (23 kDa, pI = 4.66) are resolvable by the two-dimensional gel system that was used [30–32] but did not co-immunoprecipitate with GAP-43 following DSP treatment of cells in Figure 2d or in Figures 4b, 6b or 7b. It is important to consider if a 3 hour incubation with [^35^S]-methionine and [^35^S]-cysteine is sufficient to label all of these proteins. It has been shown that spectrin [33], Rab5 effector proteins like rabenosyn-5 [31], and SNAP-25 [32] all become labeled following the incubation of isolated cells with [^35^S]-methionine for 3 hours or less. A six hour incubation was used to label the two Goα isotypes with [^35^S]-methionine in differentiated N1E-115 cells and each protein was found to have a half-life of approximately 49 hours [29], which is similar to the average half-life for total cellular protein. These results suggest that the three hour incubation period used in the present work is sufficient to label these proteins, and that their lack of co-immunoprecipitation with GAP-43 following DSP treatment of cells is not due to their being insufficiently labeled.

### 2.3. GAP-43 does not appear in the 100,000 × g pellet fractions

In Figure 3, the 100,000 × g pellet fractions obtained above from DMSO- and DSP-treated cells were analyzed by two-dimensional electrophoresis. The crosslinks were cleaved prior to electrophoresis by 2-mercaptoethanol, which was present in the sample buffer.

The same proteins are present in Figures 3a and 3b, but they are present at a greater level in Figure 3b, suggesting that crosslinking had occurred. Actin (marked with an A) is present at the highest level of any protein, both with and without DSP treatment. The migration of the proteins is altered slightly in Figure 3b because DSP neutralizes lysine residues and therefore causes a change in isoelectric points. The position of GAP-43 can be seen in Figures 2b and 2d, and based on this no trace of GAP-43 is present in Figure 3a or Figure 3b. GAP-43 was readily detected on the film after a 1 h exposure of the gel in Figure 1. A gel exposure of 4 h in Figure 3b insures that the absence of GAP-43 in Figure 3b is not due to underexposure of the gel. GAP-43 therefore did not become crosslinked to actin or to any other protein present in this pellet fraction and did not sediment with them. The fact that actin and not tubulin is the crosslinking of microtubules when applied to cells at 1 mM. At this concentration the reagent may not penetrate to the cytoskeleton, where the microtubules are located, and may be consumed by reaction with F-actin and other membrane skeleton proteins.

### 2.4. Polyclonal anti-GAP-43 yields co-immunoprecipitated calmodulin but not actin following crosslinker treatment of cells

In Figure 4, the experiment of Figure 2 was repeated using a rabbit polyclonal antibody to GAP-43. No increase in the amount of actin in the immunoprecipitate is seen when cells are treated with DSP (Figure 4b), compared with control cells treated with DMSO (Figure 4a). Calmodulin (marked with a C) again co-immunoprecipitated with GAP-43 following DSP treatment of cells (Figure 4b), but is more faintly detected than in Figure 2d. The 60 kDa protein seen in Figure 2d is not seen in Figure 4b. The higher background in Figure 4b also makes detection of the 34 kDa protein more difficult.

### 2.5. Quantitation of GAP-43 and calmodulin in immunoprecipitates

The GAP-43 and calmodulin spots were cut out of various gels, using the films as templates, followed by determination of the amount of radioactivity present. These results are shown in Table 1. The values for calmodulin have been divided by 9/4 to reflect the fact that calmodulin has 9 methionines while mouse GAP-43 has 2 methionines and 2 cysteines. The amount of calmodulin present in Figure 2d is therefore 14% of the amount of GAP-43 present. Table 1 also shows that calmodulin represented 39% of the total GAP-43 when DSP was added to lysed cells. Comparing the data for Figure 2d with Figure 2b in Table 1 shows that 65% of the GAP-43 recovered in the control immunoprecipitate is recovered in the immunoprecipitate obtained from DSP-treated cells. This at first suggested that the missing 35% would be in the 100,000 g pellet, but it is not, as shown in Figure 3b. Instead, additional GAP-43 was recovered by treating the supernatant obtained after GAP-43 immunoprecipitation with anti-calmodulin (Figure 2e). However, when this amount of GAP-43 (322 cpm) was added, the total amount of GAP-43 recovered from DSP-treated cells was still only 76% of the GAP-43 obtained from DMSO-treated cells. Table 1 shows that the recovery of GAP-43 was also not complete when polyclonal anti-GAP-43 was used, with 78% of the GAP-43 being recovered following DSP treatment of cells. Two possibilities exist to explain these results. One is that DSP has altered GAP-43 so that immunoprecipitation is not quantitative with either monoclonal or polyclonal antibodies. The other possibility is that neither antibody recognizes GAP-43 because it is crosslinked to actin or to some other protein. To distinguish between these possibilities, the experiment of Figure 2e was repeated, using anti-actin instead of anti-calmodulin.

### 2.6. Anti-actin does not yield co-immunoprecipitated GAP-43 when cells are treated with crosslinker

The experiment of Figure 5 was carried out as in Figure 2e, with antibodies added to supernatants obtained after GAP-43 immunoprecipitation.

If actin crosslinked to GAP-43 is blocking access of GAP-43 antibodies, then a polyclonal antibody to actin should recover this GAP-43. A supernatant received normal rabbit serum as a control (Figure 5a) while another supernatant received rabbit anti-actin (Figure 5b), followed by immunoprecipitation. It can be seen in Figure 5b that actin was immunoprecipitated but there is no co-immunoprecipitated GAP-43. This result gives evidence that the lack of quantitative immunoprecipitation seen in Table 1 is not due to the presence of GAP-43/actin crosslinked complexes but may be due to an alteration of GAP-43 by DSP. The lack of quantitative immunoprecipitation being due to the crosslinking of GAP-43 to some protein besides actin cannot be ruled out by the experiment of Figure 5. Other proteins that might be interaction partners for actin are not present in the immunoprecipitate. One explanation is that any interaction partners that were crosslinked to F-actin sedimented and are present in the 100,000 × g pellet seen in Figure 3b.

### 2.7. GAP-43 does not sediment with the membrane skeleton when DMSO- or DSP-treated cells are lysed in nonionic detergent

In Figure 6, labeled N1E-115 cells were treated with either DMSO or DSP and were lysed in buffer containing NP-40 substitute instead of SDS.

The lysate was centrifuged at low speed to remove nuclei and the cytoskeleton, and the supernatant was centrifuged at 100,000 × g for 1 h to sediment the membrane skeleton. Figure 6a shows that GAP-43 was immunoprecipitated from the supernatant after removal of the membrane skeleton by centrifugation. GAP-43 was therefore not associated with F-actin and did not sediment with it. Figure 6b shows that the same result was obtained if DSP was added to cells. Actin was not co-immunoprecipitated from the supernatant with GAP-43. Actin is present following DSP treatment at an even lower level than in the DMSO control. As in Figures 2 and 4, calmodulin co-immunoprecipitated with GAP-43 as a result of DSP treatment, as did proteins that are faintly present at 34 kDa and 60 kDa (marked with arrows) (Figure 6b). GAP-43 is not present in the membrane skeleton pellet obtained from cells treated with either DMSO (Figure 6c) or DSP (Figure 6d).

### 2.8. GAP-43 is crosslinked to calmodulin but not to actin if cells are lysed in nonionic detergent prior to the addition of crosslinker

In Figure 7, labeled N1E-115 cells were lysed in buffer containing 1% NP-40 substitute prior to the addition of crosslinker.

Sodium fluoride was included in the lysis buffer to inhibit the dephosphorylation of GAP-43. The nuclei and cytoskeleton were removed by low speed centrifugation, and supernatants received either DMSO as a control or DSP at a final concentration of 1 mM. After incubation for 15 min at room temperature, the unreacted DSP was quenched by the addition of glycine, and SDS was added at a final concentration of 0.1%. The lysates were then sheared through a needle and centrifuged at 100,000 × g for 1 h. Immunoprecipitation with monoclonal anti-GAP-43 was then carried out. Comparing Figure 7b (DSP-treated lysate) with Figure 7a (DMSO-treated lysate) shows that DSP treatment caused calmodulin to co-immunoprecipitate with GAP-43, as well as proteins faintly present at 34 kDa and 60 kDa. GAP-43 was not present in the 100,000 × g pellet obtained from either DMSO- (Figure 7c) or DSP-treated (Figure 7d) lysates.

Chemical crosslinking was used in this study to investigate *in vivo* if GAP-43 interacts directly with F-actin and other proteins that have been proposed, based on *in vitro* studies, to be interaction partners. Addition of DSP to living cells caused an increase in the amount of actin sedimenting at 100,000 × g following SDS lysis and the addition of nonionic detergent, but no GAP-43 was found in this pellet fraction, indicating that GAP-43 did not become crosslinked to F-actin and did not sediment with it. GAP-43 was found in the 100,000 × g supernatant, but no co-immunoprecipitated actin was seen. Following nonionic detergent lysis of cells, GAP-43 was bound to the membrane skeleton [34] and co-purified with membrane skeleton proteins [35]. In the present work, GAP-43 did not sediment with the membrane skeleton following the lysis of DMSO- or DSP-treated cells with nonionic detergent.

Although GAP-43 binds to F-actin *in vitro* [15–17], it may be prevented from doing so in the cell. Actin filaments do not directly associate with the lipid bilayer, where palmitoylated proteins are located, but are attached indirectly by binding to other proteins [36]. Filopodial actin filaments are parallel, unbranched, and linked together by fascin, and are thought to arise via the Arp2/3 complex from the meshwork of branched F-actin located in the lamellipodium [37]. GAP-43 is present on the filopodial plasma membrane as well as at sites that are not filopodial [38]. Evidence from proteasome attack suggested that GAP-43 is unfolded at physiological ionic strength [7]. If GAP-43 (226 amino acids) is in an extended conformation and is completely unfolded in the cell, its total length would be 75 nm. Given the diameter of a typical filopodium as 78 nm [39] and the fact that the N-terminal 14 residues of GAP-43 are attached to the lipid bilayer, the remaining length of GAP-43 could extend nearly the width of the filopodium. This suggests that some portions of GAP-43 might have access to filopodial F-actin. Intramolecular interactions could shorten GAP-43 and prevent this access. Interactions between the acidic C-terminal region and the basic region occur in full-length MARCKS (myistoylated alanine-rich C kinase substrate), and this prevents the protein from interacting with actin [40]. Evidence for the intramolecular binding of the acidic C-terminal region of GAP-43 to the basic calmodulin-binding region has also been obtained [41]. An alternative possibility is that the entire length of GAP-43 is adsorbed to the lipid bilayer by electrostatic interactions. In addition, GAP-43 does not leave the lipid bilayer following phosphorylation by protein kinase C [26] or when it is depalmitoylated [10]. GAP-43 would therefore not re-enter the cytosol where G-actin is polymerizing and the ability of GAP-43 to act as a capping protein for F-actin [16] would be limited by this membrane attachment. In agreement with the present results, GAP-43 could not be observed decorating actin filaments in immunostained neurons [26]. When a mutant GAP-43, in which Cys-3 and Cys-4 were replaced with glycine, was expressed in COS-7 cells, the protein showed a cytosolic distribution and did not bind to actin filaments [42]. Finally, a 70 kDa GAP-43/calmodulin crosslinked complex was observed in nonreducing gels when DSP was added to unstimulated cells [25]. When crosslinker was added following the activation of protein kinase C and the release of GAP-43 from calmodulin, an increase rather than a decrease in the level of free GAP-43 in nonreducing gels was observed [25], which implies that GAP-43, once released from calmodulin, does not become crosslinked to actin or to other proteins. Bands were detected at 120 kDa following crosslinker addition which were GAP-43-positive and calmodulin-negative, and it was suggested that these bands represent a crosslinked complex containing actin and GAP-43, but no attempt was made to identify the bands with antibodies to actin [25]. Following DSP treatment of cells, bands at 120 kDa were not observed in the nonreducing lanes of Figure 1.

Calmodulin co-immunoprecipitated with GAP-43 following DSP treatment of cells and additional GAP-43 was recovered from the immunoprecipitation supernatant with anti-calmodulin. Combining these results indicates that approximately 25% of the total GAP-43 was crosslinked to calmodulin. The fact that the remainder of the GAP-43 was not crosslinked to calmodulin is consistent with the cells being in a stimulated, differentiated state. The calmodulin crosslinking is also consistent with the fact that both unphosphorylated GAP-43 and GAP-43 phosphorylated by protein kinase C can exist in the same stimulated neuron, with phosphorylated GAP-43 predominating at the growth cone and unphosphorylated GAP-43 being present in the cell body and proximal axon [43]. Also co-immunoprecipitating with GAP-43 were proteins at 34 kDa and 60 kDa. The 34 kDa protein formed a long streak, suggesting a range of isoelectric points. This and its molecular weight suggest that it is not Goα. The 60 kDa protein has a pI more acidic than GAP-43 but less acidic than calmodulin.

What is the biological significance of GAP-43 binding to calmodulin in these cells? If the function of GAP-43 is to bind and sequester PI(4,5)P_2_, as proposed [26], it is not clear why some of the GAP-43 is bound to calmodulin. It is clear that in a growing neuron, all regions of the plasma membrane are not equivalent, with protein kinase C phosphorylating GAP-43 in some regions and not others [43]. Similarly, GAP-43 could be bound to PI(4,5)P_2_ in some regions and to calmodulin in other regions of the plasma membrane. In cultured hippocampal neurons that were not treated with any agent to activate protein kinase C, approximately 50% of the total GAP-43 could be crosslinked by DSP to calmodulin [25]. These cells are producing growth cones in the absence of these agents, so the release of GAP-43 from calmodulin that was observed when these agents were added suggests that protein kinase C has general access to most of the GAP-43 in the growing neuron but it is not activated in all regions. The purpose of the GAP-43/calmodulin interaction may still be as proposed [23], in that GAP-43 sequesters calmodulin in certain regions and releases it following GAP-43 phosphorylation by protein kinase C.

## 3. Conclusions

The lack of quantitative recovery of GAP-43 by immunoprecipitation following DSP treatment of cells leaves open the possibility that GAP-43 becomes crosslinked to other proteins in the cell. The results tend to rule out GAP-43/actin interactions in these cells. It is clear from the present study that extensive crosslinking of GAP-43 to cytoskeletal and membrane skeletal proteins does not occur in living N1E-115 cells, suggesting that GAP-43 function in these cells may derive in part from its interaction with the lipid bilayer.

## 4. Experimental Section

### 4.1. Materials

N1E-115 cells (CRL-2263) were obtained from the American Type Culture Collection, Manassas, VA. Mouse monoclonal anti-β-galactosidase and rabbit antibody to GAP-43 (whole molecule as antigen) were from Affinity Bioreagents, Golden, CO. Mouse monoclonal anti-GAP-43 (clone 91E12) was from Millipore. Protein A-agarose and protein G plus agarose were from Santa Cruz Biotechnology, Santa Cruz, CA. Calmodulin Sepharose 4B was from Amersham Biosciences. Cell culture grade water, pH 3.5–10 ampholines, Hybri-max sterile DMSO, mouse monoclonal anti-calmodulin, rabbit anti-actin, Kodak Biomax Light film, 2-mercaptoethanol, rabbit skeletal muscle actin, and all cell culture grade chemicals used in preparing modified Puck’s D1 solution were from Sigma. Nonidet P 40 (NP-40) substitute was from Fluka. Dithiobis (succinimidyl propionate) (DSP) was from Pierce. HPLC grade DMSO was from Sigma-Aldrich. Fetal bovine serum, dialyzed fetal bovine serum, and penicillin/streptomycin solution were from Invitrogen. Sodium pyruvate (100 mM) was from Mediatech, Herndon, VA. Dulbecco’s modified Eagle’s medium (DMEM), Met, Cys-free DMEM, and TRAN35S-Label were from MP Biomedicals, Solon, OH. Complete Mini protease inhibitor tablets were from Roche. All materials used in gel electrophoresis, except those given above, were obtained from Bio-Rad, Hercules, CA. Soluene 350 tissue solubilizer and Enlightning autoradiography enhancer were from Perkin-Elmer.

### 4.2. Cell culture and differentiation

N1E-115 cells were grown in Dulbecco’s modified Eagle’s Medium (DMEM) containing 4.5 g/L glucose, 4 mM L-glutamine, 3.7 g/L sodium bicarbonate, and no pyruvate, and supplemented with 10% fetal bovine serum and 1% penicillin/streptomycin at 37 °C in a humidifed atmosphere of 10% CO_2_ in air. The cells were plated on untreated 60-mm cell culture dishes, and 24 h later each dish received 5 mL of differentiation medium, which consisted of the above DMEM supplemented with 1% fetal bovine serum, 1% penicillin/streptomycin, 1 mM sodium pyruvate, and 2% Hybri-Max DMSO. Each dish received 5 mL of fresh differentiation medium every 24 h and the cells were used in crosslinking experiments after 4 days.

### 4.3. Labeling of differentiated cells

This was performed as described n the literature [44]. Differentiated N1E-115 cells in 60-mm dishes had their medium replaced with 5 mL of DMEM (minus Met and Cys) supplemented with 1% dialyzed fetal bovine serum, 1% penicillin/streptomycin, and 2 mM L-glutamine, followed by incubation at 37 °C for 30 min. Each dish then received 0.1 mL (1.05 mCi) of TRAN35S-Label, which yielded a final concentration of 0.2 mCi/mL. The cells were incubated for 3 h at 37 °C.

### 4.4. Addition of crosslinker to living cells

The labeled cells were washed three times at room temperature with 5 mL of Krebs-Ringer’s-Hepes solution (KRH), which was prepared as described [25]. KRH contained 125 mM NaCl, 4.8 mM KCl, 2.6 mM CaCl_2_, 1.2 mM MgSO_4_, 25 mM HEPES (pH 7.4), and 5.6 mM glucose. Dishes 1 and 2 then received 4.8 mL of KRH containing 0.2 mL of DMSO, while dishes 3 and 4 received 4.8 mL of KRH containing 0.2 mL of freshly prepared 25 mM DSP in DMSO. This yielded a final DSP concentration of 1 mM and a final DMSO concentration of 4%. Incubation was carried out for 15 min at room temperature.

### 4.5. Lysis of crosslinker-treated cells in SDS

Following removal of the DMSO and DSP solutions in KRH, each dish received 0.1 mL of 1% SDS in 103 mM glycine (pH 7.5). The glycine quenched unreacted DSP. Each dish then received 0.9 mL of NP-40 lysis buffer, which contained 20 mM sodium phosphate (pH 7.4), 1% NP-40 substitute, 150 mM NaCl, and 1 mM EDTA. Ten milliliters of this solution received one Complete Mini protease inhibitor tablet immediately before addition to the dishes. Each lysate was then sheared by passage six times through a 23 gauge needle. The lysates were centrifuged at 30,000 rpm (100,000 × g) for 1 h at 4 °C in a Beckman SW50.1 rotor. The pellets were dissolved in sample buffer for two-dimensional electrophoresis. The supernatants were used for immunoprecipitation as described below.

### 4.6. Lysis of crosslinker-treated cells in nonionic detergent and isolation of the membrane skeleton

The membrane sleleton was isolated as described [45]. Each dish containing either the DMSO or DSP solution in KRH received 0.5 mL of 0.1 M ethanolamine (pH 7.7) and 0.5 mL of 0.113 M glycine (pH 7.5) to quench unreacted crosslinker. After 15 min at room temperature, the solutions were removed and each dish received 0.9 mL of NP-40 lysis buffer. The lysates were centrifuged for 4 min at 14,000 rpm in a microcentrifuge. The pellets, containing nuclei and cytoskeleton, were discarded and the supernatants were centrifuged at 4 °C and 30,000 rpm (100,000 × g) for 1 h in the SW50.1 rotor. The pellets, containing the membrane skeleton, were solubilized in sample buffer for two-dimensional electrophoresis. Each supernatant received 9.1 μL of 10% SDS and was sheared six times through a 23 gauge needle. The supernatants were then used for immunoprecipitation.

### 4.7. Addition of crosslinker to cell lysates

Labeled cells on 60-mm dishes were lysed by the addition of 0.9 mL of NP-40 lysis buffer. The nuclei and cytoskeleton were removed by centrifugation, and supernatants 1 and 2 each received 36 μL of DMSO while supernatants 3 and 4 each received 36 μL of 25 mM DSP in DMSO. After incubation at room temperature for 15 min, each tube received 100 μL of 2% SDS in 0.113 M glycine (pH 7.5). After 15 min, each lysate was passed six times through a 23 gauge needle and was centrifuged in the SW50.1 rotor at 30,000 rpm (100,000 × g) for 1 h at 4 °C. The supernatants were used for immunoprecipitation as described below.

### 4.8. Immunoprecipitation of GAP-43, calmodulin, and actin

The 100,000 x g supernatants from cells lysed in either SDS or NP-40 substitute received 1 μL of mouse monoclonal anti-β-galactosidase as a control or 2 μL of mouse monoclonal anti-GAP-43 (clone 91E12), or 7 μL of mouse monoclonal anti-calmodulin solution. Each tube then received 20 μL of protein G plus agarose suspension, followed by rotation overnight at 4 °C. The agarose pellets were washed three times with 1 mL of 20 mmol/L sodium phosphate (pH 7.4), 1% NP-40 substitute, 0.1% SDS, 150 mM NaCl, 1 mM EDTA. The pellets were suctioned dry and the bound proteins were eluted by the addition of 100 μL of sample buffer for two-dimensional electrophoresis. A rabbit polyclonal antibody to GAP-43, which was obtained using full-length GAP-43 as immunogen, was also used to immunoprecipitate GAP-43. The 100,000 × g supernatants received 2 μL normal rabbit serum as a control, 9 μL of rabbit polyclonal anti-GAP-43 solution or 2 μL of rabbit anti-actin serum. Twenty microliters of protein A-agarose suspension was added to each sample followed by rotation overnight a 4°C. The pellets were washed three times as above and were treated with 100 μL of sample buffer for two-dimensional electrophoresis.

### 4.9. Gel electrophoresis

One-dimensional electrophoresis was performed as described [46]. Electrophoresis was carried out at room temperature for 18 h at 11 mA per gel. The gels were fixed, stained, dried, and exposed to film as described below for two-dimensional gels. Two-dimensional electrophoresis was carried out as described [47], except that the isoelectric focusing gels were run at 400V (constant voltage) for 22 h at room temperature, only pH 3.5–10 ampholines were used, and NP-40 substitute was used instead of NP-40. The first dimension gel mixture contained 5.5 grams urea, 1.33 mL of a solution containing 28.38% (w/v) acrylamide and 1.62% (w/v) bisacrylamide, 2 mL of 10% (w/v) NP-40 substitute, 1.97 mL water, 0.5 mL of a 40% (w/v) solution of pH 3.5–10 ampholines, 10 μL of a 10% (w/v) solution of ammonium persulfate, and 7 μL of TEMED. The sample buffer was prepared by mixing 5.7 grams urea with 2 mL 10% (v/v) NP-40 substitute, 0.5 mL of a 40% (w/v) solution of pH 3.5–10 ampholines, and 0.5 mL of 2-mercaptoethanol. The solution was then brought to 10 mL with water. The second dimension consisted of a 12% polyacrylamide gel that was run at 11 mA per gel for 18 h at room temperature. Molecular weight standards were myosin (200 kDa), β-galactosidase (116.25 kDa), phosphorylase b (97.4 kDa), albumin (66.2 kDa), ovalbumin (45 kDa), carbonic anhydrase (31 kDa), trypsin inhibitor (21.5 kDa), lysozyme (14.4 kDa), and aprotinin (6.5 kDa). For fluorography, fixed and stained gels were agitated in 180 mL of Enlightning autoradiography enhancer for 30 min. The gels were then dried, placed on Kodak Biomax light film, and stored at −75 °C for 1 h to 6 days. The films were developed using an automatic film processor (AFP Corp., Elmsford, NY).

### 4.10. Determination of radioactivity in gels

The dried gels were overlaid with the x-ray film, and radioactive spots were cut out of the gel with a razor blade. The spots were rehydrated with 0.1 mL of water for 1 h at room temperature. Each vial then received 1 mL of a 9:1 (v/v) mixture of Soluene 350 tissue solubilizer and water, followed by incubation at 50 °C for 6 h. Each vial received 50 μL of glacial acetic acid and 10 mL scintillation fluid, followed by determination of radioactivity in an LKB Wallac model 1209 Rackbeta liquid scintillation counter.

## Figures and Tables

**Table 1.** Quantitation of GAP-43 and Calmodulin in Immunoprecipitates.

	GAP43	CaM	%
Figure 2b DMSO	2967	0	0
Figure 2d DSP	1933	278	14
Figure 2e antiCaM	322		
Figure 4a DMSO	791		
Figure 4b DSP	619		
Figure 7b DSP	1650	643	39

A representative result of four experiments is shown. Radioactivity was extracted from the gels for the designated proteins. All values are in cpm.
